# “When I became older, I started having to manage that more myself”—Experiences of adolescents with food allergies: A qualitative study

**DOI:** 10.1111/pai.70048

**Published:** 2025-02-21

**Authors:** Meg O'Sullivan, Margaret Curtin, Rachel Flynn, Juan Trujillo, James O'Mahony

**Affiliations:** ^1^ University College Cork Cork Ireland; ^2^ Cork University, Hospital Cork Ireland

**Keywords:** adolescents, food allergy, qualitative, self‐management, transition

## Abstract

**Background:**

It's often caregivers or healthcare professionals' experiences that are studied in the field of allergy, but the adolescents' perspective is crucial to develop interventions that support them in areas they find most challenging. This study aims to explore adolescents' experience of managing food allergies, particularly how they navigate the transition from parental management to self‐management.

**Methods:**

This is an interpretive descriptive qualitative study. Semi‐structured interviews were conducted with ten adolescents with food allergies aged 12–16 years. Reflexive thematic analysis was conducted. The Reflexive Thematic Analysis Reporting Guidelines were used.

**Results:**

Four themes were generated, (1) belonging—seeing me, (2) not knowing (3) taking responsibility—“So I guess when I became older, I started having to manage that more myself” and (4) variation in coping strategies. These describe adolescents need for belonging, that their peers acknowledge their food allergies without making them feel like a burden. Adolescents understanding of essential food allergy information was lacking, with some unaware of this lack of knowledge. Adolescents were conscious of the need to take over responsibility from their parents, although this could be difficult. Finally, how adolescents coped with all these aspects of their food allergy could be seen as avoidant due to high anxiety, minimizing of risk, or adaptive, where adolescents are aware of and mitigate risks appropriately, without avoiding life experiences.

**Conclusion:**

Adolescents find the transition of responsibility from their parents difficult. A trusted source of allergy information is required, aimed at adolescents, covering both physical allergy management and psychosocial content.

**Key message**

This study highlights that knowledge gaps exist even when adolescents seem confident in their knowledge on allergy management. Adolescents need support in taking over responsibility for their allergy management from their parents, particularly in areas they find challenging. Coping strategies need to be discussed with adolescents with food allergies, and adaptive coping strategies encouraged. A trusted source of allergy information is required, aimed at adolescents, covering both physical allergy management and psychosocial content.

## 1 INTRODUCTION

Food allergies are estimated to affect approximately 4% of children worldwide.^1^ The potential for children to outgrow their allergy is influenced by several factors, including the allergen in question, with approximately 80% of children outgrowing a cow's milk or hen's egg allergy, compared to 10%–20% of those with peanut or tree nut allergies.^2^, ^3^ Thus, food allergies remaining in adolescence tend to persist into adulthood.^3^

This is therefore an important period for promoting self‐management, as habits developed in adolescence are more likely to be carried forward throughout life.^4^ As with all chronic health conditions, adolescence is a period of transition in which management for the condition is gradually taken over by the child from the parents, in preparation for adulthood. A recent Danish study, which examined being a teenager with food allergy through the perspective of young adults aged 18–23, revealed that with hindsight, the participants found that period in their lives “overwhelming”.^5^

While the risk for fatalities due to food allergy‐related anaphylaxis is low, adolescence is known to be the most dangerous life stage.^6^ This is hypothesized to be due to reasons such as the reduced parental supervision of adolescents, as they naturally spend more time with peers, lower risk perception, and poor adherence to recommended management.^6^ However, a trend towards decreased social participation was identified among Irish adolescents with food allergies, possibly due to heightened anxiety surrounding risk management, which is concerning for their quality of life and social development.^7^ Therefore, supporting adolescents to get the balance right in this transition period is crucial.

It has been acknowledged that adolescents need interventions to support all aspects of self‐management, which can have positive impacts on outcomes such as knowledge and quality of life, but these are lacking for adolescents with food allergies.^8^, ^9^ Before developing such interventions, healthcare professionals should carefully consider adolescents' experiences from their first‐person perspective, to best understand and support them appropriately. Conversely, in pediatric research, it is often caregivers or healthcare professionals' experiences and opinions that are sought, with children and adolescents' voices neglected.^10^ Including adolescents in healthcare research reflects the intended use of Article 12 of the United Nations Convention on the Rights of the Child—childrens' and adolescents right to not just have the space and opportunity for their voice to be heard, but also for this “voice” to have an appropriate audience and influence.^11^, ^12^

Research in the field of allergy has historically been “almost exclusively quantitative.”^13^ ^(p1118)^ Since this publication, a qualitative meta‐aggregation of teen experiences with food allergy was conducted, and a limited number of studies were identified despite no search date limit, which were found to be mostly of poor methodological quality.^14^ This meta‐aggregation recommends further, philosophically consistent research be done, “particularly using methods suited to understanding meaning and experience”.^14^ ^(p2535)^

This study aims to explore adolescents' experience of managing food allergies, particularly how they navigate the transition from parental management to self‐management. This will be of distinct use to healthcare professional readers (audience), in informing how to best support these patients in everyday clinical practice and in the development of specific interventions for this adolescent group (influence).

## 2 METHODOLOGY

### 2.1 Study design

An interpretive descriptive design, as described by Thorne,^15^, ^16^ was deemed most suitable as description a way of understanding experiential questions that maintain a focus on clinical relevance for nursing practice. Interpretive description is aligned to a constructivist paradigm, which assumes that a persons' reality is actively constructed based on their experiences and thoughts.^17^, ^18^ Thus, participants perspectives are contextually bound and ultimately subjective, and knowledge is co‐constructed between participants and researchers.^18^, ^19^ That is to say, the researchers' prior clinical experience influences and enriches the entire research process, which underscores the “researcher as instrument” viewpoint.^15^, ^20^

### 2.2 Participants and context

Eligible participants were children aged 12–16, diagnosed with at least one food allergy and prescribed an Adrenaline Auto‐Injector (AAI). Participants were recruited from the Pediatric Allergy service of the largest Health Services Executive (publicly funded) hospital in Ireland, which sees approximately 40% of children with allergies in the country.^21^ Pediatric allergy services in Ireland are acknowledged to be under‐resourced.^22^ Typically, patients are seen every 1–2 years for brief clinic appointments which often include skin prick testing and/or blood testing. Education is typically discussed by a member of the nursing or medical team, and handouts given, such as with allergy action plans. Potential participants were identified and recruited by examining upcoming clinic lists, and the contact parent or guardian of eligible patients were sent by post an invite letter, a consent‐to‐contact form, and a stamped addressed envelope. Interested parties outlined whether they preferred to be contacted by phone, email, or post and were further contacted in this manner. Ten adolescents participated, including one pair of twins. Four were male and six were female, with ages ranging from 12 years and 3 months to 16 years and 1 month. The concept of information power was utilized in determining when to stop recruiting, which indicates that the more information the sample holds, relevant for the study aim, the less participants are needed.^23^ This involves additional considerations including the specificity of the sample and the quality of the dialogue.^23^

### 2.3 Dataset generation

Semi‐structured interviews were conducted by the first author, a female pediatric nurse who had no previous relationship with the participants. The interview guide was designed to elicit adolescents' experiences of managing their own allergies in different settings, such as at home, school, and social settings, the process of taking on more responsibility for this as they age, and perceived support needs (see Appendix S1). Throughout the process, the precise wording and emphasis of questions were tailored as per the interviews' flow and adolescents' reactions, as “flexibility is key” in interviewing this age group.^10^ ^(p11)^

Adolescents were given the option of either an online interview on Microsoft Teams or in person, at the university associated with the hospital which they attend. The majority of adolescents chose online, with two interviews conducted in person. Interview duration ranged from 10 min and 10 s to 34 min, with a mean interview duration of 18 min and 16 s. Interviews were conducted between November 2023 and March 2024, at a time and date suited to the adolescents. The absence of parents during the interviews helped create an atmosphere conducive to openness,^24^, ^25^ though adolescents were given the option to have their parents present if desired, but none chose to do so.

Interviews were video recorded if the adolescent chose an online interview or audio recorded if they chose an in‐person interview. Interviews were transcribed verbatim by the first author, and for online interviews, the transcription feature of MS Teams was used as a base. Transcripts were then anonymised, with pseudonyms given.

### 2.4 Ethical considerations

Ethical approval was obtained from the Clinical Research Ethics Committee of the Cork Teaching Hospitals. Two participation information leaflets were given to families, one for parents or guardians and another developmentally appropriate one for adolescents. Adolescents signed assent forms in addition to their parent or guardians consent form and were given the opportunity to ask questions. Before the interview, the adolescents understanding and willingness to participate were again verbally discussed and confirmed, to reduce mere compliance with parental desire for the adolescent to participate,^10^, ^24^ No compensation was offered for participation.

### 2.5 Data analysis

Reflexive thematic analysis, as detailed by Braun and Clarke,^26^ was employed to analyse the data. The familiarization phase was performed by the first author through conducting and transcribing the interviews and reading through the transcripts. Co‐authors contributed to the analysis at each subsequent phase. This occurred through discussion with frequent meetings. Initially, coding was performed iteratively using NVivo software, capturing both semantic and more latent meanings. When generating initial themes, the analysis moved to manual methods on a mind map board. Themes were then developed further, with some becoming combined. Naming and ordering themes was important to the overall story of the analysis. Lastly, the act of writing up the themes and choosing which data extracts were used to illustrate these finalized the analytic process.^20^

### 2.6 Enhancing trustworthiness

Various techniques were employed to ensure overall rigor or trustworthiness of the study, the confidence and value in its' findings.^27^ For instance, throughout the entire process, an online reflexive journal was maintained by the first author. This was used as a prompt and an opportunity to reflect on and engage with both the data, and the impact of the personhood of the researcher upon the process.^28^ For instance, adolescents level of engagement with an interviewer can be a barrier to obtaining rich data.^24^ The first authors relative youth, and reflexivity in maintaining a friendly, conversationalist manner while picking up on participants interests, helped to reduce any sense of power imbalance and foster an honest and open dialogue. Thus, adolescents were more forthcoming with their experiences than anticipated, and consequently, richer data were generated.

In addition, there is congruency between the study methodology of interpretive description and analytical method of reflexive thematic analysis, which both highlight that data do not speak for themselves, and advocate that the researchers' interpretation is an essential component.^15^, ^20^ The Reflexive Thematic Analysis Reporting Guidelines were used to guide reporting (see Appendix S2).^29^ These recently published guidelines have new recommendations, such as incorporating relevant existing research and theory into the reporting of themes to add contextualized meaning, rather than solely in the discussion.

## 3 THE ANALYSIS

**FIGURE 1:**
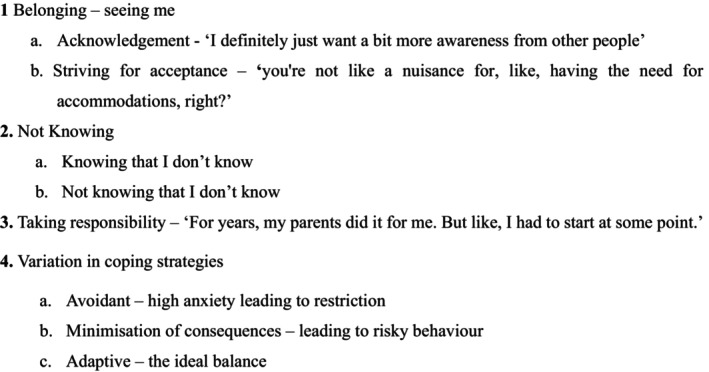
Thematic structure.

From the analysis, four main themes were generated, namely belonging—seeing me, not knowing, taking responsibility, and variation in coping strategies (see Figure 1). Adolescents articulated the need for belonging—that others acknowledged their food allergies and accepted what they needed to do to keep themselves safe, without making them feel like a burden. This, adolescents felt, was due to wider societies poor understanding of food allergies. However, adolescents with food allergies own understanding of essential food allergy information was lacking, with a varying range of insight into this knowledge deficit. Nonetheless, adolescents were conscious of the need for them to take over responsibility for the management of their food allergies from their parents, with some roles felt to be more difficult than others. Finally, how adolescents coped with all these aspects of their food allergy mirrored a previously identified theory of three different ways of coping.^30^

### 3.1 Belonging—Seeing me

The first theme concerns a sense of belonging. Belonging is a natural human need, whether it is within family, friends, the school community, or the wider public. Acknowledgement, which can be the first step towards feeling accepted, was a common thread throughout the data. A desire for peer acceptance is especially prominent among adolescents.^31^

#### 3.1.1 Acknowledgement—“I definitely just want a bit more awareness from other people”

There was a sense that as the adolescents matured, and particularly since they made the transition to secondary school, that there was a lack of recognition or consideration of their allergy, leading to feeling unimportant:

I know not everyone has an allergy, so like, they're not like, making it a priority for everyone to know about it. Christine, f15

The jump from primary to secondary school was a stark change from an environment where “the whole class knew really, I suppose” to typically bigger schools with rotating teachers, leading to an anonymity of those with food allergies, where now, “like not everyone in school knows I have an allergy”. As a result, “you definitely have to be more careful in secondary school than you would have had to be in primary school.” Students with food allergies often felt left out because their condition was overlooked:

Maybe like the teachers might be giving out like free sweets or something, you know. And then I just couldn't have them. Conor, m15

While subtle, these incidents of social isolation are widespread and contribute to feelings of alienation.^32^ In addition to feeling more included, safety was a concern for adolescents in the school environment. Adolescents believed that peers being informed and capable of providing support in the event of an allergic reaction would help them feel safer:

I think I'd be more confident if I had someone around me that knew about my allergy and like, knew how to use my pens [AAI]…But if I didn't have anyone like that around me, I think I'd be more like, panicky and I wouldn't be as confident like in using it. Christine, f15

Overt physical bullying using an adolescents' allergen, while less common than verbal bullying, is known to have a profound psychological impact.^33^ One adolescent described an incident where a boy with a peanut allergy in her school was being bullied using peanut‐containing products to taunt him. She attributed this behavior to a lack of understanding of his classmates:

Like, as much as the school tried to prevent this from happening, it was just really difficult to like, stop a student from just putting a [nut containing chocolate bar] in their bag and then like opening it…. I don't think those students understood the severity of what he was going through each time. And then I know that's gave him some severe anxiety to even come in to school. Emma, f16

On the other hand, having a “supportive and understanding” friend group was noted to nurture a sense of relatedness and belonging. Adolescents with this experience tended to feel “lucky to have friends like that”. However, these tended to be adolescents such as Ava, f15, who spoke of informing and teaching their friends about their allergy; “All my friends know how to use it [AAI pen] because they've been shown.” Furthermore, a connection with a peer with a similar condition has been widely recognized as beneficial,^34^ as highlighted by Niamh, f12: “One of my best friends is allergic to nuts as well… we definitely relate a lot.”

Given that adolescents felt more comfortable in the setting of peers who were familiar with allergies, education of the wider community was seen as key to more general awareness and understanding. This was desired particularly in schools, targeted at their peers in a general conversation about allergies, rather than them specifically:

I know this isn't like an option that's been given, but is there any possibility healthcare providers can go around to schools to give talks?…. definitely having that education of it can actually really impact someone in so many different ways. Like it's definitely, like, necessary. Emma, f16

#### 3.1.2 Striving for acceptance—“you're not like a nuisance for, like, having, the need for accommodations, right?”

Differences are particularly difficult at the adolescent stage, with almost universal desires to “sort of like to fit in with my friends and stuff”. However, what adolescents seemed to find most difficult was not necessarily having to take certain precautions that marked them as different to others, but rather having to constantly explain these:

It's sometimes hard at parties… it's just trying to explain my allergies really. To like, the parents. Robert, m12

Adolescents feared they were a burden and worried about other peoples' reactions to their needs. For example, Emma, f16, describes feeling “guilty” when she was out with her friends and couldn't eat at a food establishment they wanted to go to as she was allergic to fish and they deep‐fried everything in the same oil. She felt “horrible for bringing it up”, and although her friends agreed to all go to a different restaurant, she still felt it was “not a pleasant experience”. Some adolescents discussed sometimes just avoiding advocating for their allergy needs due to being “too embarrassed”, for example by pretending they didn't want any food instead of drawing attention to themselves by asking questions. Alternatively, Ava, f15, reflected that “sometimes it's embarrassing, but I always say it because I have to be, like, safer than sorry.”

Participants were very conscious of others' attitudes towards accommodations they requested. For instance, Ava, f15, felt the need to ask a waitress “does it ever annoy you, that like, asking questions?”, when rightfully enquiring about potential allergens in her order. Also, Anita, f15, spoke about the vast contrast between the experiences of attending two different teenage discos, one where the organizers were proactively accommodating of medical needs, and another where she felt unwelcome:

There was one we went to and it was really well run. Like there was a MedVan [non‐emergency transport vehicle, staffed by healthcare professionals] there, and now the one we go to now is like, a bit awkward. And just like, they're nearly offended we have to like, give them [AAI pens], do ya know what I mean?

In contrast, Conor, m15, highlighted the value of one person going out of their way to include him without making it a “big deal”. He was attending an Irish language camp away from home, and “the Bean an Tí [woman of the house], she was very good. Like she got even, like [ice cream brand] that I could have, like vegan [ice cream brand] that didn't have milk and eggs in them.”

For adolescents with allergies to feel a true sense of belonging and acceptance, their peers and the wider public need to have a better awareness and understanding of allergies. Alas, this may be a tall ask, as even among these adolescents with allergies, their own understanding and knowledge had proved to be lacking, as further outlined in the next theme.

### 3.2 Not knowing

**FIGURE 2:**
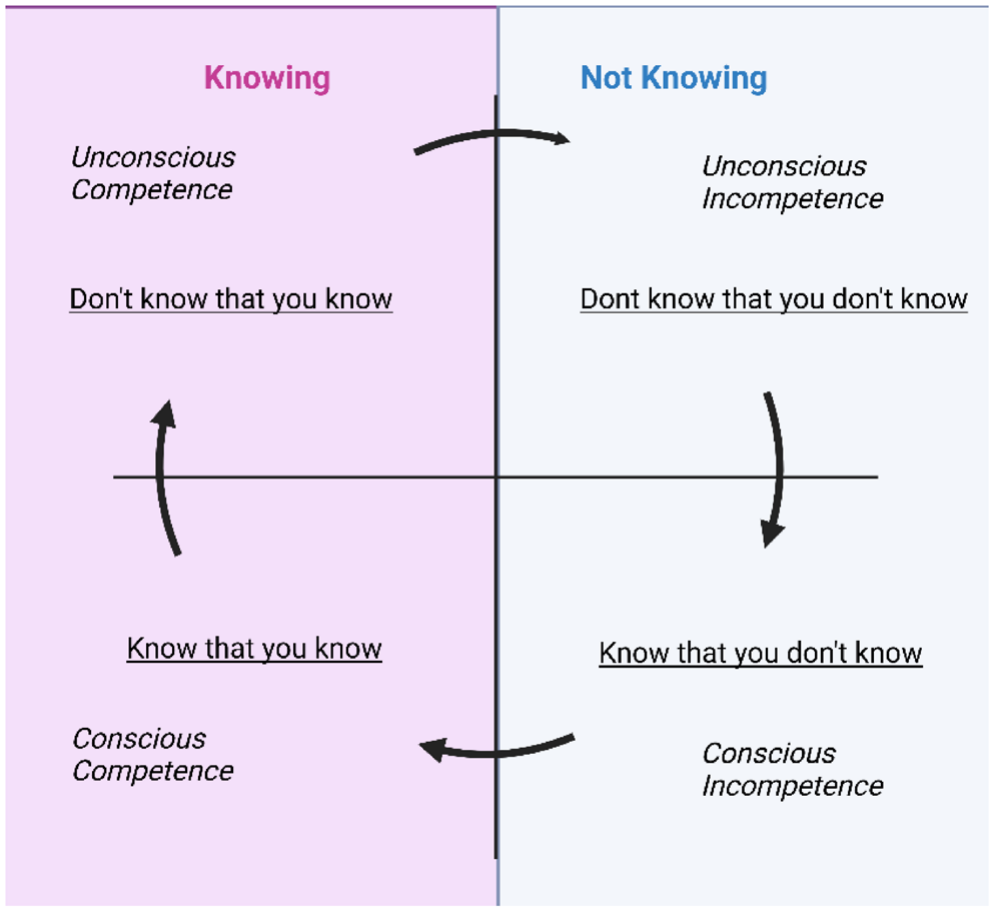
Illustration of conscious competence model.

The lack of allergy knowledge among adolescents, despite most having been allergic since childhood, was strikingly common throughout the data. This “not knowing” was divided into two distinct groups, reminiscent of the conscious competence model^35^, ^36^: those who “know that they don't know” and those who didn't realize their gaps in knowledge. The first group openly discussed aspects of food allergy management they were unsure about or used the interview to ask questions—akin to conscious incompetence. Conversely, some adolescents appeared confident in their knowledge but unknowingly mentioned incorrect practices or declared misconceptions, demonstrating they “don't know that they don't know,” or their unconscious incompetence (see Figure 2).

#### 3.2.1 Knowing that I don't know

Many adolescents were very conscious of gaps in their food allergy management knowledge, that they “wouldn't really know much about it”. This was particularly evident surrounding anaphylaxis management, as Ellen, f14 outlined; “Yeah, ‘cause I don't really understand like how they [AAI pens] work and things…I don't really know when I'm supposed to give it… And like, also the second one, how do I know if I'm supposed to give the second one?’ Thankfully anaphylaxis is not a routine occurrence in adolescents” daily lives, but as a result of this inexperience, their ability to respond appropriately can be compromised, with dangerous consequences.^6^ Many adolescents also used the interviews as a means of asking broader allergy‐related questions to a healthcare professional, actively seeking information:

Do many people grow out of it, their allergies, after the age of twelve? Robert, m12

Recognizing previous mistakes and learning from these experiences was also identified. For example, Ava, f15, reported that she delayed recognizing an allergic reaction due to not fully understanding the signs and symptoms of anaphylaxis, mistakenly attributing them to her allergic rhinitis, a common concomitant atopic disease:

So one of the main symptoms I got was an itchy throat. And I thought that was normal because I have hay fever and it's just kind of like that.

Adolescents also reported various sources for accessing information, including “Google”, “Youtube”, and asking their parents questions. However, it has been demonstrated that online sources of food allergy information can be dubious.^3^, ^37^ Also, parental knowledge may not always be completely accurate, or their answers as rounded or nuanced as allergy healthcare professionals would like their patients to receive:

But I think one thing I get confused on is like the ‘may contain’, ‘cause like Mom always tells us, like if we're picking something up and it just says ‘may contain nuts', she's like you should be fine, they're just putting that on it as a precaution. Christine, f15

A trusted resource for food allergy information was desired, as outlined by Anita, f15:

So it would be good to have a source, an easier source.

#### 3.2.2 Not knowing that I don't know

More concerningly, some adolescents revealed incorrect aspects of allergy management they were performing, in a very casual way, with the implication that they did not fully appreciate that it was ill‐advised or understand why. For instance, John, m14, would only inform restaurant staff of his allergy “if it was kind of like a dessert, yeah. But not really,” as “most of the menus have at the bottom, like if they have peanuts or not.” Other examples included relying on past experience with a certain product, as noted previously.^38^

well, if I've eaten stuff before in the past, and I know I can eat it, I don't like, I don't bother to read it again. Ava, f15

Some adolescents seemed to believe that if they had not personally experienced anaphylaxis thus far, that they did not have the potential to, and that after taking antihistamines, they'd always be “fine in about an hour.”

An interesting opportunity for misconceptions to develop appeared to be when healthcare professionals were speaking to and teaching parents, in the presence of a younger child who seemingly isn't paying attention, but who is vaguely listening and picks up pieces of information which they then put together incorrectly. For example, Paul, m13, had a food challenge previously, “like I once had an appointment with like peanut, I tried a quarter of a peanut”. His parent would have been given the standard post challenge information, including not exercising that evening, “they like mentioned don't do that much exercise or running after” and educated on co‐factors that could make a reaction worse, like sleep deprivation. However, he appeared to believe that tiredness in itself could cause a reaction, “If you really get tired like you could swell up I think”, and therefore had some worry about a sleepover planned for the following night. Similarly, he likely heard that being ill could make a reaction worse. But he appeared to have taken that to mean that a reaction could “cause you infection”. If not corrected, these misconceptions from childhood can be carried on into adolescence and beyond.

Overall, while there are certainly gaps in participants' food allergy management knowledge, the day‐to‐day basics are *known* by adolescents. However, taking responsibility for *doing* these is a large part of the transition to adulthood journey.

### 3.3 Taking responsibility—“For years, my parents did it for me. But like, I had to start at some point”

Taking over responsibility for their allergy management from their parents was seen as necessary by adolescents, but challenging, as recognized previously.^39^ Ava, f15, remarked that she's “more independent” now, “whereas when I was younger, I was more reliant on my mother and father for that sort of stuff.” Adolescents were conscious that the safety net of their parents' vigilance is now not always with them:

Like, obviously I have the responsibility of carrying them [AAI pens] around because I'm not with my parents outside anymore, so it's a little bit more pressure I suppose to remember and to just be aware of what I'm (pause). Like where I am and what's there, you know? Anita, f15

This transition to self‐management was an ongoing process. However, where adolescents were on that continuum of responsibility varied, with factors like maturity of the adolescent and familiarity of the task affecting what adolescents took on independently, or still needed help with. The mainstays of food allergy management are avoiding ones' allergens and being prepared to deal with an allergic reaction due to accidental exposures of these.^3^ Therefore, it is unsurprising that reading food labels and carrying emergency medication such as Adrenaline Auto Injectors (AAIs) were the main “tasks” participants focused on mastering. Some adolescents took on these aspects of management diligently, such as Niamh, f12:

I bring them [AAIs] everywhere, even if there's gonna be no food, just to have them, just in case…. I check every packet I see.

However, many adolescents appeared to be more episodic in their adherence. For some, the reason seemed to be due not believing that these behaviors were always necessary, with a laissez faire attitude and vague language such as “probably”, “sometimes”, and “usually”. For others, the reason for poorer compliance seemed to be for not wanting to accept the identity of the condition and a discomfiture around perceived difference:

Because like, when we were younger, whatever like, going out around with our parents and stuff, like they, Mom would usually like carry the pens [AAI] or whatever in her bag. But I, like now, like going around like we've to carry them ourselves and like (pause). I wouldn't, I'm not gonna say like sometimes I feel embarrassed but like, sometimes I just don't wanna be carrying them around with me like. Christine, f15

However, forgetfulness seemed to be the most common barrier, with many adolescents intending to follow their management plan, but remaining reliant on their parents to prompt them. It did stand out that participants had insight into these lapses, and were actively trying to improve:

But when I'm like, going out, well sometimes they're [AAI's] in Mum's handbag… I try to remember to bring them places. But I forget sometimes because it's just kind of hard to remember…. Normally Mum reminds me, but I need to like, remember myself. Ellen, f14

Similarly, adolescents were cognisant of the potential for error when independently ensuring their food is allergen‐free. Christine, f15, recalled how her last‐minute check caught her mistake:

Like for example, the other night I was in swimming and they have like a vending machine or whatever. So I like went over, I got a bar from it and I like, I felt like I'd had it before. I was like, oh, I've had this before, but then I like checked it, just before I ate it, and I was like, wait, I can't have this.

However, an aspect of food allergy management that many adolescents struggled with was communicating with food industry staff to inform them of their allergies. When asked who would tell the waiter or waitress about their allergies, Robert, m12 replied “Most of the time my mother does it”, with John, m14 similarly responding, “Yeah, Mom would do that. Mom or Dad would ask.” Those who had begun to take on this role acknowledged their own accountability:

So, I guess I did have to take more responsibility in actually going away and finding out these things and looking at the, like, venue and talking to the servers a bit more… Emma, f16

Reflecting on the overall process of shifting responsibility, some found it to be daunting:

And I suppose as well like, it's a bit harder to manage it sometimes myself, ‘cause like as well, Mom would have looked after what I was eating and stuff when I was younger. And now I have to like, be more careful when I'm just with my friends and stuff, because, like, it's me, I have to look after myself. Yeah, so I would say it's like, it's a bit more harder, like now that I'm older. Christine, f15

Some adolescents viewed this transition more as a series of goals to achieve, such as Paul, m13, set for himself small achievable next steps; “Like I might start getting my own food ready for myself. Or organise myself, like pack my bag.” For others, their increasing age and experience had given them more confidence in managing their allergies:

When I was younger I couldn't, like I didn't know what I could and couldn't have. But now like, if I'm getting something I'd know if I could have it or not, and it's much easier. Conor, m15

Looking to the future, there was a sense of optimism that, with increased knowledge, they would continue along the path of increasing responsibility, to eventually become a young adult independent in managing their allergies:

Probably the older I get, the more, like, mature I'd be about it, and like, careful. Like, I have a lot of experience with it now, I know what I can have, I know what I can't have. So I don't think it would get harder or more difficult to manage. Because you know, I'll have, like, a better understanding of it. Anita, f15

How an adolescent takes responsibility for their food allergy management can by affected by how they cope with their allergy, as further explored in the following theme.

### 3.4 Variation in coping strategies

Adolescence is a turbulent time in and of itself. Navigating adolescence while also dealing with food allergies is undeniably challenging. Although allergic reactions are fortunately not a daily occurrence, the management needed to avoid these is continual. Many adolescents reported a sense of having to be always “switched on”, such as John, m14; “Like, you'd always be wondering if there was peanuts in it.” This constant vigilance can be tiring and permeates all aspects of life:

like you wouldn't think a fish allergy would affect your daily life, but surprisingly, it does. In like, a load of respects. Emma, f16

There is always a risk, as emphasized by Ava, f15:

it's just it's always like that ‘what if’ is always in my head… it's always kind of a worry.

Having a food allergy is something that adolescents accept and deal with in different ways. From participants accounts, it was striking how these seemed to reflect the coping strategies identified by Dunngalvin et al.^30^ in their theoretical framework of developmental pathways in food allergy, namely avoidant, minimisation and adaptive strategies.

#### 3.4.1 Avoidant—high anxiety leading to restriction

An avoidant coping strategy is associated with high levels of anxiety and low self‐efficacy. Some adolescents reported avoiding places and situations that would likely cause them to be in the presence of their allergen as a strategy to manage risk and the resulting anxiety, like Robert, m12; “No, we don't really go to restaurants.”. Emma, f16, reported a self‐imposed ban on attending family gatherings altogether, as a relative would eat fish, and she was not comfortable to be in the same room as her allergen:

And like, even when I'm, if I'm out with my family, one of my uncles is pescetarian. He refuses to give up having that lack of protein every dinner. So he's eating fish every dinner, even though he knows I have an allergy. So I have to avoid those kinds of social events.

Thus, her heightened anxiety caused decreased participation in social activities.

Christine, f15, appeared to struggle with her “allergy identity”, finding even practicing with trainer AAI pens intimidating; “Umm, I suppose about using the pen [AAI], like when I was younger, my Mom and Dad used to get like the fake ones [trainer AAI]. I used to hate it ‘cause I was like this is so scary. Like I don't want to have to do this.’ She similarly used avoidance as a strategy to manage others” perceptions;

Before I was like, fully confident with my friends, I was kind of like, I don't know, like anxious to talk about it because I was like, what are they gonna say now.

#### 3.4.2 Minimisation of consequences—leading to risky behavior

On the other end of the spectrum, minimisation coping strategies such as knowingly partaking in “risky” behaviors were also observed, although least commonly. Examples included purposefully not carrying AAIs or eating foods that may contain their allergen. The “minimisation” aspect came from asserting that these behaviors were not “a big deal” and did not have the potential for serious consequences. For instance, Paul, m13, while talking about a bake sale in school in which classmates had brought in goods baked at home, said he would eat them if the other child said they did not contain his allergen – even in an unsure manner, having no information about cross‐contamination; “Like it depends. I might ask are there peanuts in this, and then they'd be like, am no. And then I might just take them.” Among a set of twins, one sister reflected on the differences between them on how they coped with their respective food allergies, and felt she was more “careful” than her sister, and how her sister “would be a bit more, not willing, but she'd like, test it.”

#### 3.4.3 Adaptive—the ideal balance

Reassuringly, adaptive coping strategies seemed to be most commonly used, which lies at the centre of the continuum and refers to positive coping strategies, associated with emotional resilience. Such adolescents seemed to make an active choice to “get on with it”:

I don't really, like, get stressed over it. I just like, look at it and see, like ‘oh too bad I can't eat it’. I just eat what I can eat…. You can have the same fun, like, you know, it doesn't matter. Paul, m13

Conor, m15, similarly focused on the positives, and the improvements he has seen over the years:

It's actually not too bad like. Like I'd say if I was a teenager five years ago with allergies it would have been harder. Like there's a lot of options and stuff now for food… and like most of the ingredients, most of them, they be highlighted.

This adaptive style of coping does not mean the adolescent does not see the risk or does not feel the anxiety, or even venture into “toxic positivity” where they are not acknowledging the difficulty of their situation, but rather they are consciously dealing with it in a resilient manner:

So it's kind of like, just trying to avoid it at all costs is like, difficult, but it's doable. Emma, f16

Similarly, Anita, f15, acknowledges that although some aspects of allergy management can be uncomfortable, she has the intrinsic motivation to do the correct thing herself anyway, because she recognizes its importance:

It's kind of like, well, it's either that [bring AAIs to the disco] or, you know, the risk of not having them. So yeah, it is a little bit awkward, but it has to be done because you know, it's too dangerous.

Emma, f16, felt that this ability to “feel the embarrassment and do it anyway” is something that she had learned over time, and that reinforcing this with younger adolescents could be helpful:

I think that like, it just needs to be explained to the point of view where it's not embarrassing. It's just a part of you that like, you can't actually control. It's just your body's response to certain foods.

## 4 DISCUSSION

This study explored adolescents' experience with food allergies, particularly the changes associated with this time in their lives. Adolescents struggled with finding a sense of belonging, not knowing enough about aspects of food allergy management, taking over responsibility from their parents, and finding a balance of healthy coping measures.

Given where adolescents spend much of their time, awareness of allergies in schools and among friends is particularly vital. However, adolescents lacked confidence in their safety in this environment. This reflects a survey of 200 adolescents and young adults with food allergy which found that only 11% felt their classmates would know what do in an emergency.^40^ Although overt bullying was mentioned, most issues were due to exclusion rooted in lack of awareness, rather than intentional. Adolescents wanted their classmates to be taught about allergies, similar to a study which found 68% of adolescents believed education of their friends would make living with food allergy easier.^41^ However, adolescents do not want this education to be about them specifically, as adolescents already deal with “unwanted attention”.^14^ ^(p2540)^ Given that participants who had taught their friends about their food allergy management felt “lucky” and more content with their friend group, perhaps these positive experiences had more to do with education and preparation than simply good fortune. This reflects the European Academy of Allergy and Clinical Immunology (EAACI) transition guidelines, which recommend that healthcare professionals encourage adolescents to let their friends know about their allergy and how to manage emergencies.^42^

In management of food allergies “a high level of education is needed to maintain safety”.^3^ ^(p47)^ However, adolescents were lacking in the level of knowledge about their food allergies that would be expected of them, sometimes without awareness of this knowledge gap. This is concerning, as insufficient knowledge about potential elicitors of reactions, anaphylaxis‐related symptoms and treatment represent one of the leading barriers to successful self‐management among teenagers.^6^ Ultimately, this leads to non‐use or delayed use of adrenaline in cases of anaphylaxis, which is the leading risk for fatality.^43^ Healthcare professionals need to support adolescents knowledge, with the aim to move them from the “not knowing” to the “knowing” side of the of conscious competence model (Figure 2), through being made familiar with the necessary information and skills, and practicing until it becomes “unconscious” or second nature.

As adolescents age, they must assume an increasing level of responsibility for their food allergy management, with the ultimate goal of becoming independent, capable young adults.^44^ If this gradual process of responsibility shifting from parents to adolescent is not achieved smoothly, it could result in preventable adverse reactions.^45^ However, as our findings reiterated, many adolescents may find this transition difficult. Similar to this study, Keohane et al.^46^ determined that adolescents were still reliant on their parents for certain food allergy management behaviors, such as asking about allergens in restaurants and carrying AAI. Annunziato et al.^45^ found just 25% of adolescents reported taking responsibility for explaining their allergy to others themselves, without input from their parents. As the EAACI transition guidelines demonstrate, healthcare professionals should focus on areas of management where adolescents are not confident.^42^

However, it is not only the physical health of patients' healthcare professionals should consider—the emotional wellbeing of adolescents with food allergies is also crucial. The minimisation coping strategy group reflects a study which found adolescents and young adults with food allergy whose behaviors were highest risk, felt less concern about their allergy, despite having more recent reactions.^41^ This is the group that healthcare professionals typically are concerned about, appropriately. Surprisingly though, the avoidant coping strategy group was more common. This reflects an Irish study which found that of those with food allergies, adolescents were three times less likely to eat in food establishments than younger children.^7^ From the outside, it can seem like taking all possible measures to avoid risk is a positive thing, but when extreme, this can seriously hinder adolescents' development and wellbeing. As emphasized by Crealy & Byrne,^7^ a major goal of allergy healthcare professionals should be to empower adolescents with food allergies to participate fully in life, similar to their peers. This is also reflected in the EAACI transition guidelines, which recommend aiming for better adolescent participation in social events.^42^ Thus, healthcare professionals should support adolescents to be aware of and mitigate risks appropriately, but not avoid important life experiences.

### 4.1 Strengths and limitations

This research was conducted with methodological congruency as a core criterion, which adds trustworthiness to the findings. Although the period of transition to self‐management is recognized to include young adults up to the age of 25,^42^ this study included adolescents with an upper limit of 16 years. This was largely for practical reasons, as pediatric allergy services in Ireland discharge patients at 16 years, with no local adult allergy service to recruit from. However, ensuring adolescents were recruited from an allergy service, with diagnosed food allergy and prescribed AAI was an important strength of this study. Self‐report of food allergy is generally acknowledged to result in an overestimation of prevalence and include intolerances,^3^ and therefore lead to dilution of the true experience of adolescents with food allergies. As demographic questionnaires were not administered to adolescents, age and biological sex are the only demographics available. This is a limitation, as other factors such as gender, age at diagnosis, race/ ethnicity and socioeconomic status may have been helpful in adding context to the analysis. As with all research of this nature, the sample was motivated to take part and may therefore be different in some way to adolescents with food allergies who did not take part.

## 5 CONCLUSION

This qualitative study provides insight into adolescents with food allergies experiences. Many adolescents find the transition of responsibility from their parents difficult, with certain aspects such as communication with restaurant staff causing particular problems. Adolescents should teach their friends about their allergy management, both to feel better understood and safer. Adaptive coping should be encouraged, where adolescents have emotional resilience and are aware of and mitigate risks appropriately, but not avoid important life experiences due to excess anxiety. Healthcare professionals should be conscious of aspects of living with food allergies that adolescents typically find challenging, and proactively ask about and discuss these in routine clinical interaction in a compassionate manner. A trusted source of allergy information is required aimed at adolescents, covering both physical allergy management and psychosocial content. Future research should consider adolescents with food allergies experiences and challenges in developing interventions to support their transition to self‐management.

## AUTHOR CONTRIBUTIONS

**Meg O'Sullivan:** Conceptualization; methodology; data curation; formal analysis; project administration; writing – original draft. **Margaret Curtin:** Conceptualization; supervision; writing – review and editing. **Rachel Flynn:** Methodology; conceptualization; supervision. **Juan Trujillo:** Conceptualization; resources; methodology; supervision; writing – review and editing. **James O'Mahony:** Conceptualization; methodology; formal analysis; supervision; writing – review and editing.

## FUNDING INFORMATION

This research was supported by a PhD scholarship from the Catherine McAuley School of Nursing and Midwifery, University College Cork, Cork, Ireland.

## CONFLICT OF INTEREST STATEMENT

The authors have no conflict of interest to declare.

### PEER REVIEW

The peer review history for this article is available at https://www.webofscience.com/api/gateway/wos/peer‐review/10.1111/pai.70048.

## Supporting information


Appendix S1.

Appendix S2.
