# Supplementary material for: “When I became older, I started having to manage that more myself”—Experiences of adolescents with food allergies: A qualitative study
Source: Pediatr Allergy Immunol. 2025 Feb 21;36(2):e70048. doi: 10.1111/pai.70048 (PMC11844234; doi:10.1111/pai.70048)
Supplement: Supplementary file 1 — Appendix S1. [file PAI-36-e70048-s001.docx]

**Semi-structured interview schedule for adolescents aged 12-16 years**

1. Tell me about your experience of managing your food allergies now that you are a teenager?
2. At home
3. In school and social settings
4. Have you had any issues taking more responsibility for managing your allergies as you get older? Are you worried about this for the future?
5. Do you feel you need more support from your healthcare team? In what aspects?
6. Education – like understanding food allergies and how adrenaline works,
7. Training - skills like how and when to use your adrenaline auto-injector, how to communicate your allergy to staff when eating out,
8. Monitoring – food labels and keeping track of any reactions,
9. Emotional support - coping with allergies and issues like stress or bullying, communication with friends and family,
10. Transition support - preparing to move to adult centred healthcare, understanding financial resources,
11. Any other aspects.
12. How best could we deliver that support do you think, bearing in mind limitations of the service and your own schedule?*
13. Would you be open to what we call ‘telehealth’, which is remote healthcare using for example videoconferencing or a mobile application?
14. Would you prefer support to be individual or in groups?
15. Would you prefer support to be one or several sessions?
16. Would you use such a support if it was available?

*Footnote: data on preferences for self-management support is not analysed in this paper.
